# A comparison of transcatheter aortic valve prosthesis platforms: Myval, Sapien, and Evolut in severe symptomatic aortic stenosis and low-moderate risk patients

**DOI:** 10.2478/raon-2025-0046

**Published:** 2025-10-27

**Authors:** Matjaz Bunc, Klemen Steblovnik, Simon Terseglav, Jana Ambrozic, Mojca Bervar, Ljupka Dimitrovska, Miha Cercek, Ana Kovac, Patricija Pleskovic, Polonca Kogoj, Zlatko Fras, Miha Sustersic, Bojan Vrtovec

**Affiliations:** 1 Clinical Department for Cardiology, University Clinical Centre Ljubljana, Ljubljana, Slovenia; 2 Institute for Pathophysiology, Faculty of Medicine Ljubljana, Ljubljana, Slovenia; 3 Faculty of Medicine Ljubljana, Ljubljana, Slovenia

**Keywords:** transcatheter aortic valve implantation, aortic stenosis, real-world comparison

## Abstract

**Background:**

This article compares the real-world performance and safety of the three transcatheter aortic valve implantation (TAVI) platforms: Myval, Sapien, and Evolut in patients with severe symptomatic aortic stenosis and low to moderate surgical risk.

**Patients and methods:**

Between September 2019 and September 2023, 1053 TAVI procedures were performed in the University Medical Centre Ljubljana, Slovenia. We used propensity-score match analysis to compare the Myval, Sapien, and Evolut platforms. 180 patients were enrolled in the propensity-score matching study, 60 for each platform. The study endpoints included haemodynamic outcomes compared to baseline, in-hospital clinical safety outcomes, and all-cause mortality at 30 days and one year.

**Results:**

Changes in peak aortic valve velocity, mean aortic gradient, effective orifice area, and left ventricular ejection fraction were comparable between the platforms. After propensity score matching (tri-match), the rates of stroke (3.4% *vs*. 3.4% *vs*. 0.0%, p = 0.548), life-threatening bleeding (1.7% *vs*. 1.7% *vs*. 1.7 %), periprocedural myocardial infarction (3.3% *vs*. 0.0% *vs*. 0.0%, p = 0.330), postprocedural permanent pacemaker implantation rate (11.9% *vs*. 10.2% *vs*. 15.0%, p = 0.719), all-cause mortality at 30 days (3.3% *vs*. 5.0% *vs*. 3.3%; p = 1.000) and at 1 year (8.3% *vs*. 8.3% *vs*. 10.0%, p = 0.934) were comparable between the Myval, Sapien, and Evolut series, respectively. 2 cases of moderate paravalvular regurgitation were reported, one in Myval, and one in Sapien series.

**Conclusions:**

The tri-match analysis of the real-world aortic stenosis patients with low to moderate surgical risk treated with the Myval, Sapien, and Evolut series showed comparable performance, safety, efficacy, and survival.

## Introduction

Transcatheter aortic valve implantation (TAVI) has become the preferred treatment option for older patients with severe aortic stenosis (AS) in all risk categories, with the expansion of therapeutic indications being supported by real-world data.^1–4^ In the elderly population, a longer life expectancy alters how outcomes are evaluated. Once-acceptable rates of paravalvular regurgitation (PVR) or permanent pacemaker implantation (PPI) may need improvement.^5^

The growing range of transcatheter heart valves (THVs) aims to limit the risks associated with TAVI and thus improve clinical outcomes. The newly developed TAVI devices limit the risks associated with previous-generation devices, such as needing a new permanent pacemaker or significant residual aortic regurgitation after TAVI.^6^ However, as we consider more and more variables and patient characteristics, it is becoming clearer that real-life direct comparisons are essential for the personalised decision-making process.

Balloon-expandable valves (BEVs) and self-expanding valves (SEVs) are widely used THVs in clinical practice. Supra-annular SEVs have larger effective orifice areas (EOA) and lower gradients. SEVs can also be repositioned during implantation to gain the best outcome. BEVs have a lower risk of requiring permanent pacemaker implantation (PPI) and paravalvular leaks comparable to SEVs. The Myval THV (Meril Life Sciences, India) is a novel BEV that enables precise anatomy-sizing of the device with 1.5 mm diameter increments and 9 different valve sizes. The Sapien 3 THV (Edwards Lifesciences, California, USA) has a cobalt-chromium frame with bovine pericardial tissue leaflets and an outer skirt. It is known for its low-profile delivery system and has been accepted for AS patients in all risk categories. Evolut THV series (Medtronic, Minneapolis, USA) are SEVs with nitinol frames and are approved for severe AS patients in all risk groups. Sapien and Evolut series have consistently shown good clinical outcomes and are a well-known treatment option for severe symptomatic aortic stenosis.7,8 They have 10-year performance that is comparable to SAVR.^9^ The Myval series has been shown to be safe and effective in clinical trials and real-life data. It provides another option for people with aortic stenosis who are at high or intermediate risk for traditional severe AS surgery, but long-term durability still must be proven.^10–13^ The Landmark trial showed that the Myval THV series was non-inferior to the Evolut and Sapien series for the primary composite endpoint at 30 days.^14^ Although there are some direct and indirect comparisons of Myval, Sapien, and Evolut series valves in different combinations, the amount of comparative data for all three platforms is still limited.

In this study, we investigated haemodynamic performance (maximal aortic blood velocity, residual gradient, EOA, and PVR), safety according to the Valve Academic Research Consortium-3 (VARC-3) criteria^15^, and 30-day and 1-year allcause mortality of the Myval, Sapien, and Evolut series in severe AS patients with low to moderate surgical risk.

## Patients and methods

### Study design and population

Consecutive patients with severe AS on native aortic valve, a previous TAVI prosthesis, or a surgically implanted aortic valve (SAVR) were included in this study. They were at low to intermediate risk and received one of three TAVI devices (Myval, Sapien, or Evolut series) at the University Medical Centre Ljubljana, Slovenia. The clinical data of all patients who underwent TAVI between September 2019 and September 2023 were prospectively collected in a registry database. All three devices were available to the institution’s heart team, and operators were free to choose which specific THVs to use according to computer tomography angiography (CTA) measurements and other patients’ characteristics.

The study was conducted in accordance with the principles of Good Clinical Practice and tenets of the Declaration of Helsinki and was approved by the National Medical Ethics Committee of the Republic of Slovenia (No.: 0120-315/2024-2711-3).

### Study devices

From the Sapien platform only Sapien 3 devices were used in this study. The device is a BEV. The valve consists of a cobalt-chromium frame, three bovine pericardium leaflets, and a polyethylene terephthalate (PET) skirt to minimise PVR. The valve is available in 20 mm, 23 mm, and 26 mm sizes and is compatible with a 14Fr expandable sheath, while the 29 mm size is compatible with a 16Fr expandable sheath.^16^ The beneficial performance of Sapien-3 has also been demonstrated in the PARTNER clinical trials in intermediate and low-risk patients.^2,3^

The Evolut is a series of SEVs with nitinol frames. The design incorporates porcine pericardial supra-annular leaflets and a porcine pericardium fabric skirt. The available sizes include 23 mm, 26 mm, 29 mm, and the extra-large size 34 mm. The 23–29 mm sizes are implanted through a 14Fr-compatible delivery system or an 18Fr sheath; the larger 34 mm prosthesis is implanted through a 16Fr-compatible delivery system or a 20Fr sheath. In our study, some patients in the Evolut group received Evolut Pro and Evolut Pro+ valves, which feature an updated design aimed at reducing PVR (with the help of external tissue wrap on the frame and other features). Evolut Pro is available in 23 mm, 26 mm, and 29 mm sizes.

The Myval series is a next-generation BEV approved by Conformité Européene. The frame (nickel-cobalt) is made of continuous hexagons arranged in a hybrid honeycomb fashion. Bovine pericardium tissue with the anti-calcification treatment (AntiCa) forms a tri-leaflet valve. The lower frame cells are covered internally and externally with PET, minimising the potential for PVR. The Myval series is manufactured in conventional sizes (20 mm, 23 mm, 26 mm, and 29 mm), intermediate sizes (21.5 mm, 24.5 mm, and 27.5 mm), and extra-large sizes (30.5 mm and 32 mm). All diameters are compatible with a 14-Fr Python introducer sheath (Meril Life Sciences, India).^10^ The safety and efficacy of the Myval series in intermediate-to high-risk patients have been demonstrated in several studies.^10–13^ However, the present study focuses on low-to intermediate-surgical-risk patients.

A summary of the main differences between these THVs is shown in Table 1.

**TABLE 1.** Summary of differences between Myval series, Sapien series, and Evolut series

Device	Myval series	Sapien series	Evolut series
Images	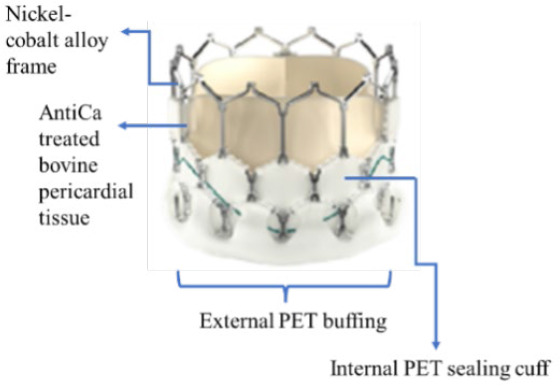	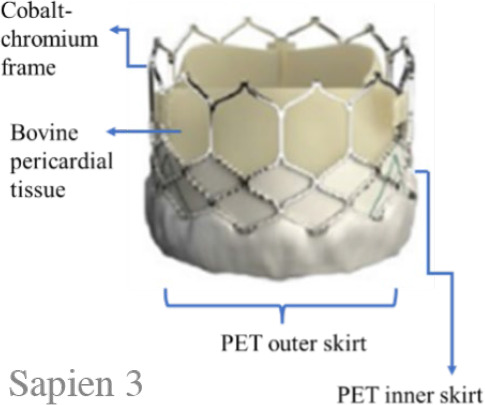	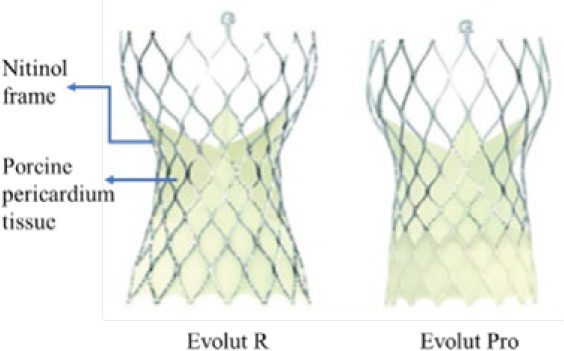
Support structure	Nickel-cobalt alloy	Cobalt-chromium alloy	Nitinol (nickel-titanium) frame
Valve structure	53% open cells on the upper half and 47% closed cells on the lower half form the hexagonal frame for the hybrid honeycomb cell design concept	Heterogeneous frame design that incorporates hexagons and diamonds. Overall, the frame consists of 5 rungs and 12 open cells, where the upper cells are larger and the lower cells are smaller	A radiopaque self-expanding nitinol support frame with a diamond cell configuration
Conventional sizes	20, 23, 26, 29 mm	20, 23, 26, 29 mm	23, 26, 29 mm
Intermediate sizes	21.5, 24.5, 27.5 mm	Not available	Not available
Extra-large sizes	30.5, 32 mm	Not available	34 mm
Valve annulus size range	18.5-32.7 mm (area derived diameter)	18.6-29.5 mm (area derived diameter)	17/18–30 mm (CT-derived diameters)
Introducer sheath	14F Python introducer sheath for all diameters (20-32 mm) Full retrievability of the undeployed Myval THV Series system	14F eSheath for 20-26mm 16F eSheath for 29mm The Sapien THV system cannot be retrieved once inside the patient	Evolut R & Evolut Pro: A 14F delivery system or an 18F sheath for 23–29 mm; a 16F delivery system or a 20F sheath for 34 mm. Evolut Pro+: 14F InLine sheath for 23-29 mm and 18 Fr InLine sheath for 34 mm Can be retrieved before full deployment
Deployment technique	The design generates a specific “zebra crossing” like pattern under fluoroscopy. This is used for position and deployment.	The design does not generate any specific pattern. Positioning is at 50% using balloon radiopaque marker.	The design does not generate any specific pattern. Positioning is controlled by radiopaque markers.

1 TAVI = transcatheter aortic valve implantation; THV = transcatheter heart valve

### Procedure

Procedures were performed in the hybrid operating room or in the catheterisation laboratory under shallow sedation in the majority of cases with local anaesthetic given at the puncture site or in general anaesthesia. Unfractionated heparin was given after all the vascular access sites were punctured. A transfemoral approach (TF) was used in all cases. THV implantation was done according to the manufacturer’s guidelines. Femoral closure devices included ProGlide/ProStyle (Abbott Vascular Devices, California, USA) and AngioSeal (St. Jude Medical, Minnesota, USA) in various ratios according to the operator’s discretion.

### Ultrasound analysis

Transthoracic echocardiograms (TTE) were obtained at baseline and at 30 days of follow-up, and the measured parameters followed the recommendations of the European and American guidelines.^17,18^ The performance of each THV was assessed by its maximum aortic blood velocity (Vmax), mean aortic gradient, effective orifice area (EOA), and left ventricular ejection fraction (LVEF). TTE was done before hospital discharge and after 30 days after hospital discharge.

### Outcomes

We looked at the following in-hospital cardiac complications: cardiac tamponade, annular rupture, valve embolisation, pericardial effusion, incorrect valve position, switching to open-heart surgery, periprocedural myocardial infarction (MI), and spontaneous myocardial infarction (MI). Other complications included access site complications, bleeding complications, and other components of post-procedure safety assessment. All the complications were assessed according to VARC-3 criteria.^15^ The analysis also included haemodynamic performance at 30 days as assessed by TTE (Vmax, mean aortic gradient, EOA, and LVEF). We compared the 30-day and 1-year all-cause mortality between the matched groups.

### Statistical analysis

Baseline characteristics were presented with descriptive statistics. Continuous variables were shown as mean and standard deviation, while nominal variables were presented as frequencies and percentages. The between-group comparisons were performed using an independent sample t-test and ANOVA in the case of quantitative variables, as appropriate. A paired t-test was used for the post-hoc analysis to evaluate the significant differences between pre-procedure and post-procedure data within each group. The Chi-square/Fisher’s exact test was used for qualitative variables, as appropriate. Propensity score matching analysis was performed using the nearest neighbour matching method to balance the study groups based on key baseline characteristics. Patients were matched based on their age, gender, Society of Thoracic Surgeons (STS), LVEF, body mass index (BMI), body surface area (BSA), New York Heart Association (NYHA) functional class, pre-procedural serum creatinine level, baseline conduction abnormalities (Right Bundle Branch Block, RBBB), and baseline atrial fibrillation at the inclusion in the study. A P-value < 0.05 was considered statistically significant. Statistical analysis was performed using R software.

## Results

**FIGURE 1.**
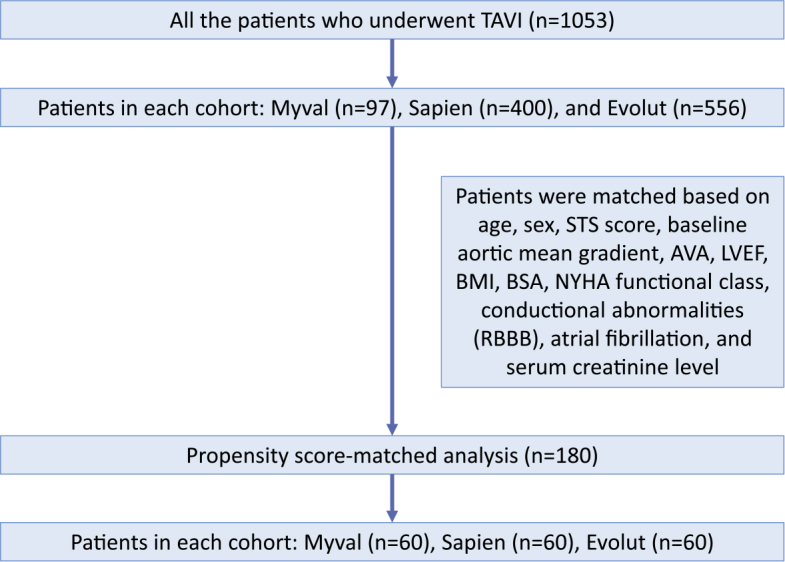
Flowchart of patient selection and propensity score matching in transcatheter aortic valve implantation (TAVI) cohorts. AVA = aortic valve area; BMI = body mass index; BSA = body surface area; LVEF = left ventricular ejection fraction; NYHA = New York Heart Association; RBBB = Right Bundle Branch Block; STS = Society of Thoracic Surgeons

A total of 1053 patients who underwent TAVI were included in the analysis. Out of 1053 TAVI patients, 97 patients received the Myval series, 400 patients received the Sapien series, and 556 patients received the Evolut series (Table 2, Figure 1). The mean age of the cohort was 81.3 ± 6.4 years, and 51.5% of the patients were male. 878 (83.4%) of the patients had arterial hypertension. The other common comorbidities included diabetes (23.8%), pulmonary disease (13.2%), chronic kidney disease (CKD) (28.7%), ischaemic heart disease (24.1%), hyperlipidaemia (28.5%), and atrial fibrillation (24.4%) (Table 2). In terms of the surgical risk in the cohort, the mean EuroScore II score was 6.17 ± 6.74% (n = 920) and the mean STS score was 4.43 ± 4.03% (n = 830). (Table 2).

**TABLE 2.** Baseline characteristics and medical history of unmatched and matched cohorts with severe aortic stenosis who underwent TAVI with different THVs. Data availability is provided in the first row for each variable, n (%)

BASELINE CHARACTERISTICS	Overall Cohort (n = 1053)	Unmatched cohorts				Matched cohorts			
		Myval series (n = 97)	Sapien series (n = 400)	Evolut series (n = 556)	p-value* (Overall)	Myval series (n = 60)	Sapien series (n = 60)	Evolut series (n = 60)	p-value* (Overall)
**Age (Years), mean ± SD**	n = 1053 (100)	n = 97 (100)	n = 400 (100)	n = 556 (100)	0.682	n = 60 (100)	n = 60 (100)	n = 60 (100)	0.729
	81.3 ± 6.4	81.0 ± 6.5	80.7 ± 6.4	81.5 ± 6.7		81.3 ± 6.7	80.5 ± 6.6	81.5 ± 7.1	
**Sex, n (%)**	n = 1053 (100)	n = 97 (100)	n = 400 (100)	n = 556 (100)	0.009	n = 60 (100)	n = 60 (100)	n = 60 (100)	
*Male, n (%)*	542 (51.5)	52 (53.6)	209 (46.2)	311 (55.9)		29 (48.3)	24 (40.0)	31 (51.7)	0.419
*Female, n (%)*	511 (48.5)	45 (46.4)	243 (53.8)	245 (44.1)		31 (51.7)	36 (60.0)	29 (48.3)	
**BMI (kg/m2), mean ± SD**	n = 1001 (95)	n = 97 (100)	n = 377 (94)	n = 527 (95)	0.003	n=60 (100)	n = 60 (100)	n = 60 (100)	0.88 5
	27.59 ± 4.87	28.56 ± 5.31	27.99 ± 5.18	27.12 ± 4.49		28.06 ± 5.12	27.62 ± 5.14	27.83 ± 4.66	
**Body surface area (m2), mean ± SD**	n = 1001 (95)	n = 97 (100)	n = 377 (94)	n = 527 (95)	0.041	n = 60 (100)	n = 60 (100)	n = 60 (100)	0.134
	1.83 ± 0.21	1.87 ± 0.22	1.81 ± 0.21	1.83 ± 0.2		1.85 ± 0.22	1.78 ± 0.18	1.82 ± 0.21	
**Indication, n (%)**	n = 1051 (100)	n = 97 (100)	n = 398 (100)	n = 556 (100)	0.445	n = 60 (100)	n = 60 (100)	n = 60 (100)	
*Stenosis*	1038 (98.8)	96 (99.0)	391 (98.2)	551 (99.1)		60 (100.0)	59 (98.3)	60 (100.0)	1.000
*Regurgitation*	13 (1.2)	1 (1.0)	7 (1.8)	5 (0.9)		0 (0.0)	1 (1.7)	0 (0.0)	
**Etiology, n (%)**	n = 1050 (100)	n = 97 (100)	n = 399 (100)	n = 554 (100)	0.414	n = 60 (100)	n = 60 (100)	n = 60 (100)	
*Degenerative*	980 (93.3)	91 (93.8)	366 (91.7)	523 (94.4)		55 (91.7)	53 (88.3)	55 (91.7)	0.024
*Rheumatic*	3 (0.3)	0 (0.0)	1 (0.3)	2 (0.4)		0 (0.0)	0 (0.0)	1 (1.7)	
**ViV, n (%)**	67 (6.4)	6 (6.2)	32 (8.0)	29 (5.2)	0.011	5 (8.3)	5 (8.3)	0 (0.0)	0.326
**Creatinine (μmol/L), mean ± SD**	n = 995 (95)	n = 97 (100)	n = 368 (92)	n = 530 (95)		n = 60 (100)	n = 60 (100)	n = 60 (100)	
	111.95 ± 74.74	102.32 ± 68.56	105.00 ± 64.36	118.55 ± 81.70		94.58 ± 36.27	100.97 ± 54.30	111.13 ± 82.84	
**DVI, mean ± SD**	n = 704 (67)	n = 68 (70)	n = 263 (66)	n = 373 (67)	0.985	n = 48 (80)	n = 50 (83)	n = 51 (85)	0.706
	0.20 ± 0.06	0.20 ± 0.04	0.20 ± 0.08	0.20 ± 0.05		0.20 ± 0.03	0.19 ± 0.05	0.19 ± 0.04	
**Systolic pulmonary artery pressure (mm Hg), mean ± SD**	n = 675 (64)	n = 60 (62)	n = 247 (62)	n = 368 (66)	0.158	n = 42 (70)	n = 51 (85)	n = 53 (88)	0.355
	42.9 ± 13.3	40.6 ± 14.3	42.3 ± 13.3	43.7 ± 13.1		38.8 ± 12.8	41.8 ± 12.38	42.4 ± 13.3	
**Euroscore 2, mean ± SD**	n = 920 (87)	n = 96 (99)	n = 321 (80)	n = 503 (91)	0.384	n = 60 (100)	n = 60 (100)	n = 60 (100)	0.307
	6.17 ± 6.74	5.41 ± 5.30	6.05 ± 6.51	6.40 ± 7.11		4.74 ± 3.82	6.40 ± 8.23	5.7 ± 4.72	
**STS score, mean ± SD**	n = 830 (79)	n = 97 (100)	n = 284 (71)	n = 449 (81)	0.219	n = 60 (100)	n = 60 (100)	n = 60 (100)	0.359
	4.43 ± 4.03	3.78 ± 3.09	4.28 ± 3.97	4.67 ± 4.24		3.90 ± 3.65	4.64 ± 5.51	5.07 ± 4.18	
**Annular perimeter (mm), mean ± SD**	n = 949 (90)	n = 91 (94)	n = 359 (90)	n = 499 (90)	0.871	n = 56 (93)	n = 59 (98)	n = 60 (100)	0.407
	80.2 ± 35.5	79.5 ± 7.2	79.6 ± 31.3	80.8 ± 41.0		79.1 ± 6.8	77.3 ± 10.2	79.0 ± 7.2	
**Annular area (mm^2^), mean ± SD**	n = 968 (92)	n = 92 (95)	n = 368 (92)	n = 508 (91)	0.293	n = 57 (95)	n = 59 (98)	n = 60 (100)	0.774
	464.5 ± 97.6	476.7 ± 94.7	459.6 ± 88.1	465.9 ± 104.3		473.6 ± 92.3	461.5 ± 95.3	468.7 ± 85.8	
**NYHA class before, n (%)**	n = 994 (94)	n = 97 (100)	n = 367 (92)	n = 530 (95)	0.471	n = 60 (100)	n = 60 (100)	n = 60 (100)	
*1*	22 (2.2)	4 (4.1)	9 (2.5)	9 (1.7)		3 (5.0)	2 (3.3)	1 (1.7)	
*2*	205 (20.6)	17 (17.5)	75 (20.4)	113 (21.3)		12 (20.0)	15 (25.0)	14 (23.3)	0.963
*3*	653 (65.7)	68 (70.1)	245 (66.8)	340 (64.2)		40 (66.7)	38 (63.3)	41 (68.3)	
*4*	114 (11.5)	8 (8.2)	38 (10.4)	68 (12.8)		5 (8.3)	5 (8.3)	4 (6.7)	
**Aortic regurgitation before, n (%)**	n = 918 (87)	n = 84 (87)	n = 333 (83)	n = 501 (90)	0.300	n = 53 (88)	n = 60 (100)	n = 59 (98)	
*None/trace*	284 (30.9)	31 (36.9)	101 (30.3)	152 (30.3)		21 (39.6)	11 (18.3)	12 (20.3)	
*Mild*	522 (56.9)	40 (47.6)	194 (58.3)	288 (57.5)		25 (47.2)	43 (71.7)	43 (72.9)	0.032
*Moderate*	90 (9.8)	12 (14.3)	27 (8.1)	51 (10.2)		7 (13.2)	5 (8.3)	4 (6.8)	
*Severe*	22 (2.4)	1 (1.2)	11 (3.3)	10 (2.0)		0 (0.0)	1 (1.7)	0 (0.0)	
**Mitral regurgitation before, n (%)**	n = 882 (84)	n = 82 (85)	n = 327 (82)	n = 473 (85)	0.090	n = 52 (87)	n = 59 (98)	n = 58 (97)	
*None/trace*	111 (1.2)	17 (20.7)	35 (10.7)	59 (12.5)		12 (23.1)	4 (6.8)	2 (3.4)	
*Mild*	697 (75.9)	64 (78.1)	261 (79.8)	372 (78.6)		40 (76.9)	50 (84.7)	50 (86.2)	0.002
*Moderate*	69 (7.5)	1 (1.2)	29 (8.9)	39 (8.2)		0 (0.0)	5 (8.5)	6 (10.3)	
*Severe*	5 (0.5)	0 (0.0)	2 (0.6)	3 (0.6)		0 (0.0)	0 (0.0)	0 (0.0)	
**Medical history, n (%)**									
*Arterial hypertension*	878 (83.4)	88 (90.7)	327 (81.8)	463 (83.3)	-	56 (93.3)	56 (93.3)	54 (90.0)	-
*Diabetes - oral antidiabetics*	179 (17.0)	12 (12.4)	63 (15.8)	104 (18.7)	-	5 (8.3)	8 (13.3)	10 (16.7)	-
*Diabetes -insulin dependent*	72 (6.8)	7 (7.2)	22 (5.5)	43 (7.7)	-	6 (10.0)	6 (10.0)	5 (8.3)	-
*Pulmonary disease*	139 (13.2)	17 (17.5)	43 (10.8)	79 (14.2)	-	9 (15.0)	9 (15.0)	11 (18.3)	-
*CKD (eGF < 60 mL/min/1.73 m^2^)*	278 (26.4)	28 (28.9)	94 (23.5)	156 (28.1)	-	18 (30.0)	17 (28.3)	13 (21.7)	-
*CKD - dialysis*	24 (2.3)	1 (1.0)	5 (1.3)	18 (3.2)	-	0 (0.0)	1 (1.7)	1 (1.7)	-
*Hyperlipidemia*	300 (28.5)	31 (32.0)	107 (26.8)	162 (29.1)	-	18 (30.0)	23 (38.3)	19 (31.7)	-
**Cardiac history, n (%)**									
*IHD (any type of revascularization, proximal significant coronary stenosis)*	254 (24.1)	26 (26.8)	79 (19.8)	149 (26.8)	-	17 (28.3)	11 (18.3)	18 (30.0)	-
*AV block I*	94 (8.9)	8 (8.2)	23 (5.8)	63 (11.3)	-	3 (5.0)	4 (6.7)	9 (15.0)	-
*AV block II*	8 (0.8)	0 (0.0)	0 (0.0)	8 (1.4)	-	0 (0.0)	0 (0.0)	1 (1.7)	-
*RBBB*	72 (6.8)	8 (8.2)	26 (6.5)	38 (6.8)	-	7 (11.7)	3 (5.0)	4 (6.7)	-
*LBBB*	89 (8.5)	10 (10.3)	33 (8.3)	46 (8.3)	-	5 (8.3)	9 (15.0)	5 (8.3)	-
*Atrial fibrillation*	15 (1.4)	1 (1.03)	4 (1.00)	10 (1.8)	-	1 (1.67)	2 (3.3)	4 (6.7)	-
*Atrial fibrillation - slow ventricular response*	242 (23.0)	22 (22.7)	91 (22.8)	129 (23.2)	-	12 (20)	14 (23.3)	9 (15.0)	-
*Electrosystolic rhythm*	66 (6.3)	3 (3.1)	32 (8.0)	31 (5.6)	-	1 (1.67)	4 (6.7)	4 (6.7)	-

1 AV = atrioventricular; BMI = body mass index; CKD = Chronic kidney disease; DVI = Doppler velocity index; eGFR = estimated glomerular filtration rate; IHD = Ischemic heart disease; LBBB = Left bundle branch block; NYHA = New York Heart Association; RBBB = right bundle branch block; SD = standard deviation; STS = Society of Thoracic Surgeons; THV = transcatheter heart valve; TAVI = transcatheter valve implantation; ViV = valve-in-valve.

A total of 180 patients from the large cohort were included in the propensity-score matching analysis. The most important baseline parameters of this subpopulation were aligned with the overall patient population. Three groups (60 for each device) were formed using propensity score matching to ensure equivalence by the essential baseline characteristics and clinical assessments. The baseline characteristics and confirmation of the absence of significant differences between the groups can be found in Table 2.

The study compared the three THV series in a low-to intermediate-risk population of patients with severe symptomatic AS. The mean STS score was 3.90 ± 3.65% in the Myval series, 4.64 ± 5.51% in the Sapien series, and 5.07 ± 4.18% in the Evolut series (p = 0.359). The mean EuroScore II was 4.74 ± 3.82 in the Myval series, 6.40 ± 8.23 in the Sapien series, and 5.70 ± 4.72 in the Evolut series (p = 0.307). The groups did not differ significantly by NYHA functional class before TAVI. Most patients were in Class III (66.7% *vs*. 63.3% *vs*. 68.3%) or Class II (20.0% *vs*. 25.0% *vs*. 23.3%) in the Myval, Sapien, and Evolut series, respectively. Previous surgical valve replacement was reported in five patients (8.3%) in the Myval series, five patients (8.3%) in the Sapien series, and none of the patients from the Evolut series.

**FIGURE 2.**
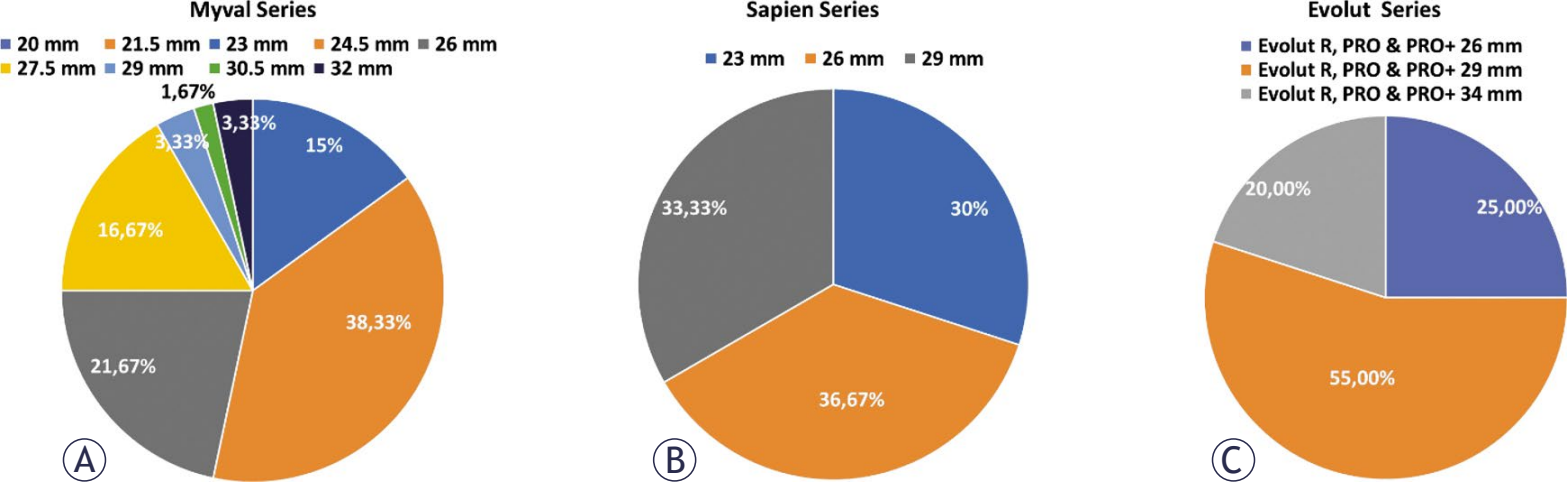
Sizes of implanted transcatheter heart valves – **(A)** Myval series, **(B)** Sapien series, **(C)** Evolut series.

The study evaluated clinical outcomes in terms of safety and performance of the device. Few patients required conversion from percutaneous to surgical closure of access site: 3 (5.0%) in the Myval series, 1 (1.7%) in the Sapien series, and 3 (5.0%) in the Evolut series. The only series in which no patient received general anaesthesia was the Myval series. Sizes of the implanted valves are illustrated in Figure 2.

Pre-dilation was performed more frequently in the Evolut series and Sapien series than in the Myval series (39 [65.0%] *vs*. 58 [37.9%] *vs*. 2 [3.3%]). Post-dilatation was more frequent in the Evolut series (29.3%) than in the Myval series (5.0%) and Sapien series (0.0%), respectively.

**FIGURE 3.**
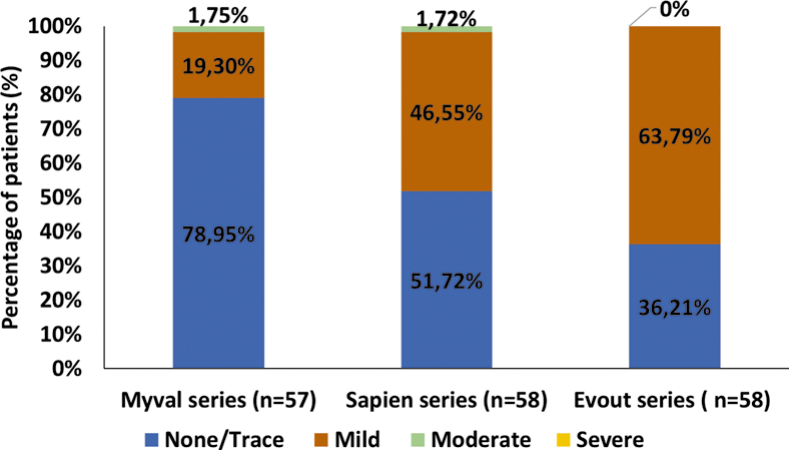
Paravalvular regurgitation after transcatheter implantation of Myval, Sapien, and Evolut series.

The groups did not vary significantly in terms of cardiac complications such as periprocedural myocardial infarction (MI) (less than 72 hours), spontaneous MI (more than 72 hours), tamponade, annular rupture, valve embolisation, improper valve position, and new pericardial effusion. The details are listed in Table 3. Regarding PVR after TAVI, most patients in all groups fell into the none/trace and mild categories (Figure 3). The new PPI rate was numerically lower in the Myval (11.9%) and Sapien (10.2%) series compared to the Evolut series (15.0%), but the difference was not statistically significant (Table 3). Significant improvement (between pre- and post-TAVI) in Vmax and EOA was observed in all three groups. It is worth noting that the proportion of valve-in-valve procedures was 8.3% in both the Myval and Sapien series, while no ViV procedures were performed in the Evolut group. Given that ViV procedures are known to be associated with higher post-procedural transvalvular gradients, this difference may have contributed to the slightly higher gradients observed in the Myval and Sapien groups. Significant improvement of LVEF was seen only in the Sapien series (p = 0.023), while in the Myval (p = 0.061) and Evolut series (p = 0.057) did not reach the level of significance. See Table 4 for further details.

**TABLE 3.** In-hospital cardiac complications in the propensity-score matched cohort. Data availability is provided in the first row for each variable, n (%)

In-hospital outcomes, n (%)	Myval series (n = 60)	Sapien series (n = 60)	Evolut series (n = 60)	p-value overall	p-value Myval series vs Evolut series	p-value Myval series vs. Sapien series	p-value Evolut series vs. Sapien series
**Cardiac complications**	n = 59 (98)	n = 60 (100)	n = 59 (98)	0.390	1.000	0.491	0.272
	3 (5.1)	6 (10.0)	2 (3.4)				
New pericardial effusion	0 (0.0)	1 (1.7)	1 (1.7)	1.000	1.000	1.000	1.000
Tamponade	0 (0.0)	4 (6.7)	1 (1.7)	0.129	1.000	0.119	0.364
Annular rupture	1 (1.7)	1 (1.7)	0 (0.0)	1.000	1.000	1.000	1.000
Valve embolization	0 (0.0)	0 (0.0)	0 (0.0)	1.000	1.000	1.000	1.000
Improper valve position	0 (0.0)	1 (1.7)	0 (0.0)	1.000	1.000	1.000	1.000
Conversion to heart surgery	0 (0.0)	0 (0.0)	0 (0.0)	1.000	1.000	1.000	1.000
Periprocedural myocardial infarction (< 72h)	2 (3.3)	0 (0.0)	0 (0.0)	0.330	0.496	0.496	1.000
Spontaneous myocardial infarction (> 72h)	0 (0.0)	0 (0.0)	0 (0.0)	1.000	1.000	1.000	1.000
**Neurological complications**	n = 58 (97)	n = 58 (97)	n = 59 (98)	0.534	0.364	1.000	0.619
	3 (5.2)	2 (3.4)	1 (1.7)				
Transient ischemic attack	1 (1.7)	0 (0.0)	1 (1.7)	1.000	1.000	1.000	1.000
Ischemic cerebrovascular insult	2 (3.4)	2 (3.4)	0 (0.0)	0.548	0.496	1.000	0.496
Hemorrhagic cerebrovascular insult	0 (0.0)	0 (0.0)	0 (0.0)	1.000	1.000	1.000	1.000
**Bleeding**	n = 59 (98)	n = 59 (98)	n = 60 (100)	0.768	0.743	0.741	1.000
	4 (6.8)	6 (10.2)	6 (10.0)				
Bleeding - minor	3 (5.0)	1 (1.7)	2 (3.3)	0.872	1.000	0.619	1.000
Bleeding - major	0 (0.0)	4 (6.7)	3 (5.0)	0.164	0.246	0.119	1.000
Bleeding - life-threatening	1 (1.7)	1 (1.7)	1 (1.7)	1.000	1.000	1.000	1.000
**Other complications**							
Acute kidney injury	n = 60 (100)	n = 60 (100)	n = 60 (100)	0.350	0.364	0.207	1.000
	1 (1.7)	5 (8.3)	4 (6.7)				
New left bundle branch block	n = 60 (100)	n = 60 (100)	n = 60 (100)	0.495	1.000	0.439	0.439
	5 (8.3)	2 (3.3)	5 (8.3)				
New atrial fibrillation	n = 60 (100)	n = 60 (100)	n = 60 (100)	1.000	1.000	1.000	1.000
	2 (3.3)	2 (3.3)	1 (1.7)				
Permanent pacemaker implantation after TAVI	n = 60 (100)	n = 60 (100)	n = 60 (100)	0.719	0.816	1.000	0.605
	7 (11.9)	6 (10.0)	9 (15.0)				
30-day mortality	n = 60 (100)	n = 60 (100)	n = 60 (100)	1.000	1.000	1.000	1.000
	2 (3.3)	3 (5.0)	2 (3.3)				
1 -year mortality	n = 60 (100)	n = 60 (100)	n = 60 (100)	0.934	1.000	1.000	1.000
	5 (8.3)	5 (8.3)	6 (10.0)				

1 MI = myocardial infarction; TAVI = transcatheter aortic valve implantation

**TABLE 4.** Comparison of haemodynamic parameters in a matched cohort. Data availability is provided in the first row for each variable, n (%)

Hemodynamic Outcomes in Matched Cohort				P value
Parameters	Cohort	Before procedure	After procedure	
Aortic Vmax (m/s), mean ± SD	Myval series	n = 56 (93) 4.3 ± 0.5	n = 58 (97) 2.1 ± 0.5	< 0.001
	Evolut series	n = 59 (98) 4.3 ± 0.6	n = 54 (90) 1.8 ± 0.4	< 0.001
	Sapien series	n = 58 (97) 4.3 ± 0.6	n = 55 (92) 2.2 ± 0.4	< 0.001
	p-value (Myval vs. Evolut)	0.780	< 0.001	
	p-value (Myval vs. Sapien)	0.908	0.355	
Aortic mean gradient (mm Hg), mean ± SD	Myval series	n = 60 (100) 47.2 ± 13.1	n = 58 (97) 11.1 ±5.4	< 0.001
	Evolut series	n = 60 (100) 46.6 ± 15.3	n = 54 (90) 6.7 ± 2.61	< 0.001
	Sapien series	n = 60 (100) 47.3 ± 14.8	n = 54 (90) 10.8 ± 3.9	< 0.001
	p-value (Myval vs. Evolut)	0.808	< 0.001	
	p-value (Myval vs. Sapien)	0.958	0.684	
AVA and EOA (cm2), mean ± SD	Myval series	n = 60 (100) 0.7 ±0.1	n = 57 (95) 1.9 ± 0.5	< 0.001
	Evolut series	n = 60 (100) 0.7 ± 0.1	n = 51 (85) 1.8 ± 0.5	< 0.001
	Sapien series	n = 60 (100) 0.6 ± 0.2	n = 56 (93) 1.7 ± 0.5	< 0.001
	p-value (Myval vs. Evolut)	0.121	0.807	
	p-value (Myval vs. Sapien)	0.068	0.026	
LVEF (%), mean ± SD	Myval series	n = 59 (98) 59.2 ± 11.1	n = 52 (87) 61.6 ± 9.9	0.061
	Evolut series	n = 60 (100) 54.8 ± 14.7	n = 52 (87) 58.5 ± 12.4	0.057
	Sapien series	n = 60 (100) 56.1 ± 15.8	n = 53 (88) 58.3 ± 14.9	0.023
	p-value (Myval vs. Evolut)	0.063	0.170	
	p-value (Myval vs. Sapien)	0.211	0.180	

1 AVA = aortic valve area; EOA = effective orifice area; LVEF = center ventricular ejection fraction. All in-group differences were significant (p < 0.05 for all within-group comparisons). Mean ± SD = Mean values and standard deviation.

**FIGURE 4.**
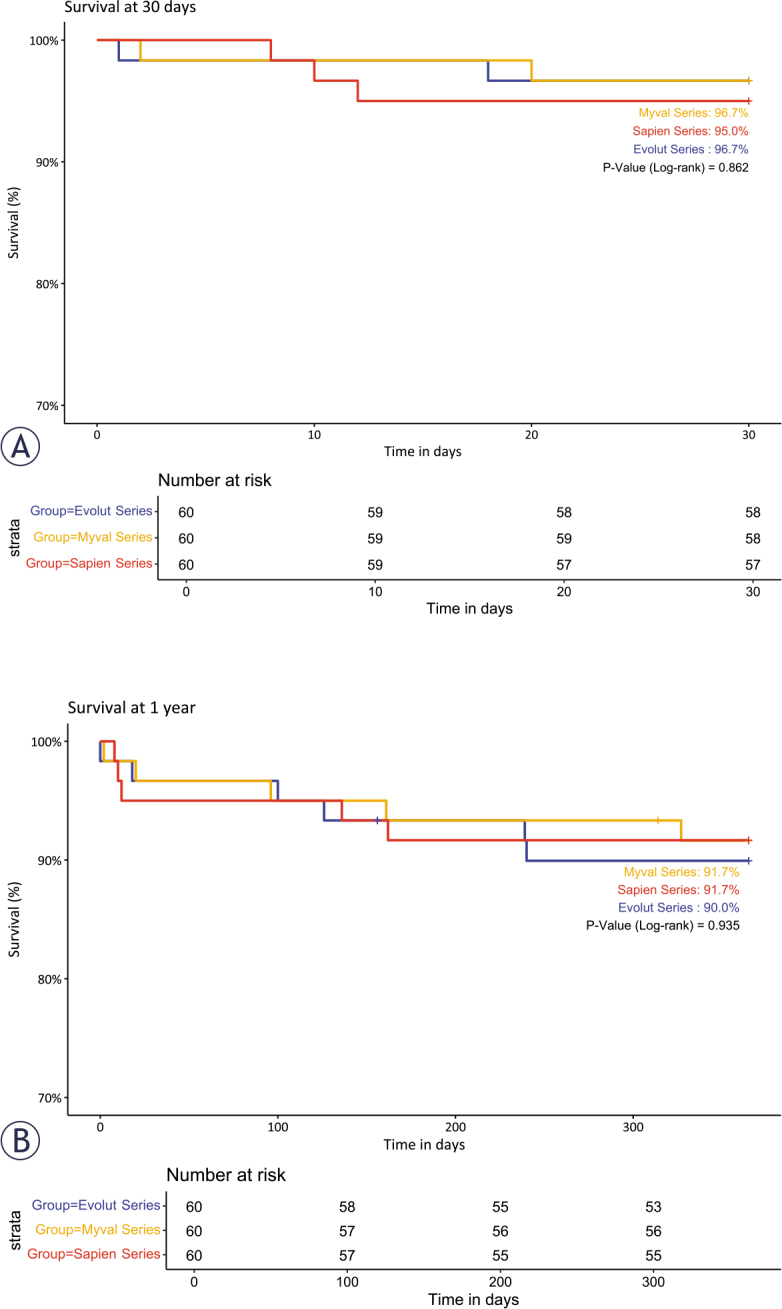
Kaplan-Meier survival curves of matched cohorts for 30 days **(A)** and 1 year **(B)** after transcatheter implantation of Myval, Sapien, and Evolut series in patients with severe symptomatic aortic stenosis.

In the matched cohort, there were two in-hospital deaths (both cardiac deaths) in the Myval series (heart failure and severe retroperitoneal bleeding after vascular complications); three in-hospital deaths (one cardiac and two non-cardiac) in the Sapien 3 series group; and two in-hospital deaths (one cardiac and one non-cardiac) in the Evolut series. Figures 4A and 4B display the Kaplan-Meier survival curves for propensity-matched cohorts at 30 days and 1 year.

## Discussion

This study was conducted to compare the real-world performance and safety of the Myval series with contemporary TAVI devices, including the Sapien and Evolut platforms. All three Evolut R, Evolut Pro, and Evolut Pro+ devices were used in this study. A previous comparison of the Myval series and the Sapien series has shown that the Myval series was favourable in terms of safety, haemodynamics, and PVR. The latter were evaluated in blinded echocardiographic assessments.^19^

Our study also showed negligible or mild PVR in the majority of cases and the absence of severe PVR in all cases. Precise sizing may also play a role in PVR reduction. The traditional diameters of TAVI prostheses were 20, 23, 26, and 29 mm. This limited-size matrix means that the nominal volume of the balloon needs to be changed so that the prosthesis fits correctly around the patient’s area-derived annulus diameter and the aortic root complex is not damaged. The Myval series’ size matrix with intermediate sizes might help to address this important issue.

Several previous studies have compared the Myval and Evolut series in patients with symptomatic severe AS. The early clinical performance and safety of Myval and the Evolut R were compared in a single-centre retrospective cohort study. 108 patients received the Evolut R THV, and 58 patients were treated with the Myval THV. The Myval series provided comparable performance to the Evolut series, and it was associated with lower rates of PPI and ≥ moderate PVR within 30 days and 6 months after the procedure.^12^ A new study using propensity score matching discovered that the two valves were comparable in terms of safety and effectiveness, with the Evolut series having a higher PPI rate. Up to 1-year of follow-up, clinical outcomes showed acceptable rates of stroke and cardiac death for both valves.^7^ In the EVAL registry, 2-year clinical and echocardiographic outcomes of TAVI were compared between the Myval and Evolut series. Both THVs showed similar 2-year clinical outcomes. Benefits from the Myval series included decreased PVR incidence and increased clinical effectiveness.^13^

In examining the outcomes associated with the Myval series, it is important to note that the SAPIEN 3 Ultra (S3U) valve has demonstrated comparable rates of death and other clinical outcomes up to 30 days post-TAVI, with both devices exhibiting remarkably low rates that align with findings from larger series involving the SAPIEN 3 (S3) valve.^3,20,21^ Notably, the S3U valve was associated with a significantly lower rate of mild PVR compared to its predecessor, with rates of 43.0% for the S3 versus 18.7% for the S3U. Interestingly, there was no significant difference in the rates of moderate or severe PVR, reported at 1.3% for the S3 and 2.7% for the S3U.^22^ These findings underscore the potential benefits of the S3U valve in achieving improved outcomes related to mild PVR, which is a critical consideration for future studies comparing it to the Myval. Discussing these comparisons may help inform best practices in valve selection and management of PVR in TAVI procedures. In another study, Stinis *et al*. compared outcomes of the SAPIEN 3 Ultra Resilia valve (S3UR) with its predecessors, the S3/S3U. After propensity matching, 10312 patients were included in each cohort. This fifth-generation BEV demonstrated significantly better haemodynamic performance compared to S3/S3U at discharge and 30 days.^23^

Frangieh *et al*. (2017) described a standardised minimalist approach for TF-TAVI using SAPIEN 3 devices through extensive operator experience at a high-volume center. The approach consists of 10 structured steps executed under fluoroscopic guidance, underscoring the importance of a collaborative heart-team strategy to optimise patient outcomes. The key findings highlight a significant reduction in intubation rates and intervention times, alongside a marked decrease in PPI from 18% to 5.6% due to optimised valve positioning. This structured framework is particularly advantageous for operators, enhancing safety and efficacy in TAVI procedures.^24^ In our study, the PPI rate for Sapien 3 was comparably lower than this study (10.17%). However, these rates can further be reduced by utilising this technique.

Four different SEVs (Evolut, Acurate [Boston Scientific, USA], Portico [Abbott, USA], and Allegra [NVT, Germany]) and one BEV (Sapien 3) were compared in an Academic European registry of 1131 consecutive patients with severe AS against Myval. The comparison was based on conduction disturbances. The results showed that Myval had similar procedural and in-hospital outcomes to the Sapien-3. It also had much lower early PPI rates than SEVs like Evolut, Portico, and Allegra. A few early conduction disruptions were associated with the Myval THVs, while some SEV choices resulted in significant variations in the PR and QRS wave-lengths.^25^ These findings are consistent with our findings from the current study. The size range of the Myval THV is wider than that of the Evolut series and the Sapien 3 series. In our matched groups, almost half of the patients used the intermediate sizes of Myval THVs. Intermediate sizes of the Evolut and Sapien series may also improve conduction disturbances and PVR.

The LANDMARK trial was a prospective, randomised, multinational, open-label non-inferiority trial that demonstrated non-inferiority of the Myval over Sapien and Evolut series for the primary combined safety and effectiveness endpoint (25% *vs*. 27%; risk difference: 2.3%, p-non-inferiority < 0.0001) at 30 days in severe AS patients.^14^ In a recent subset analysis, the LANDMARK trial has demonstrated the non-inferiority of the Myval over the Sapien and Evolut series individually.^26^ Myval series had a significantly better EOA than Sapien and a comparable EOA with Evolut series for similar THV sizes. In small aortic annuli patients, the Myval series had a comparable rate of primary composite endpoint compared to the Sapien and Evolut series.^26^

Our study has several limitations. First, the data were collected at a single centre as part of routine clinical practice. Because of this observational study design and the collection of data from real-world practice, some echocardiographic examinations and outcome assessments were not available for some of the patients, and 1-year echocardiographic data is not available. Echocardiography was performed locally as per standard practice at the study centre and not in a core echocardiographic laboratory. Second, all-cause mortality was the only outcome assessed at 1-year follow-up. While mortality is a critical outcome, the absence of longer-term follow-up data restricts a comprehensive evaluation of other important patient outcomes and the sustainability of treatment effects. Data would have been better with an echocardiographic assessment and an assessment of clinical outcomes at 1 year. Another limitation is the relatively small sample size of 180 patients, divided into three groups of 60 patients each in the propensity score-matched group, which may limit the statistical power and generalisability of the findings. Since this is a tri-match propensity score study, the results would have been more robust if the sample had been larger.
